# Vaccine Responses in Adult Hematopoietic Stem Cell Transplant Recipients: A Comprehensive Review

**DOI:** 10.3390/cancers13236140

**Published:** 2021-12-06

**Authors:** Michelle Janssen, Anke Bruns, Jürgen Kuball, Reinier Raijmakers, Debbie van Baarle

**Affiliations:** 1 Department of Infectious Diseases, UMC Utrecht, 3584 Utrecht, The Netherlands; a.h.w.bruns@umcutrecht.nl; 2 Department of Hematology, UMC Utrecht, 3584 Utrecht, The Netherlands; j.h.e.kuball@umcutrecht.nl (J.K.); r.raymakers@umcutrecht.nl (R.R.); 3 Center for Translational Immunology, UMC Utrecht, 3584 Utrecht, The Netherlands; debbie.van.baarle@rivm.nl; 4 Center for Infectious Disease Control, RIVM, 3721 Bilthoven, The Netherlands

**Keywords:** hematopoietic stem cell transplantation, vaccination, vaccine response

## Abstract

**Simple Summary:**

Patients who recently received a stem cell transplantation are at greater risk for infection due to impairment of their immune system. In order to prevent severe infection, these patients are vaccinated after their stem cell transplantation with childhood immunization vaccines. Timing of this vaccination is important in order to be effective and obtain proper immune response. Postponement of vaccination would lead to better immune response but would also cause longer-lasting risk of infection. This review describes available data on the timing of vaccination and its vaccine responses. Optimal timing of vaccination might require an individualized approach per patient.

**Abstract:**

Consensus on timing of post-hematopoietic stem cell transplantation (HSCT) vaccination is currently lacking and is therefore assessed in this review. PubMed was searched systematically for articles concerning vaccination post-HSCT and included a basis in predefined criteria. To enable comparison, data were extracted and tables were constructed per vaccine, displaying vaccine response as either seroprotection or seroconversion for allogeneic HSCT (alloHSCT) and autologous HSCT (autoHSCT) separately. A total of 33 studies were included with 1914 patients in total: 1654 alloHSCT recipients and 260 autoHSCT recipients. In alloHSCT recipients, influenza vaccine at 7–48 months post-transplant resulted in responses of 10–97%. After 12 months post-transplant, responses were >45%. Pneumococcal vaccination 3–25 months post-transplant resulted in responses of 43–99%, with the response increasing with time. Diphtheria, tetanus, pertussis, poliomyelitis and *Haemophilus influenzae* type b at 6–17 months post-transplant: 26–100%. Meningococcal vaccination at 12 months post-transplant: 65%. Hepatitis B vaccine at 6–23 months post-transplant: 40–94%. Measles, mumps and rubella at 41–69 months post-transplant: 19–72%. In general, autoHSCT recipients obtained slightly higher responses compared with alloHSCT recipients. Conclusively, responses to childhood immunization vaccines post-HSCT are poor in comparison with healthy individuals. Therefore, evaluation of response might be indicated. Timing of revaccination is essential for optimal response. An individualized approach might be necessary for optimizing vaccine responses.

## 1. Introduction

Hematopoietic stem cell transplantation (HSCT) is a promising and often the only curative option for patients with hematological malignancies [1]. Post-HSCT, the immune system is temporarily suppressed due to prior conditioning and usage of immunosuppressive medication, resulting in infection-derived complications being a major cause of transplant-related mortality [2]. Furthermore, pre-HSCT established immunity against vaccine-preventable diseases might be diminished through transplantation. To avoid infections, preventive strategies such as antibiotics, antiviral and antifungal prophylaxis and post-HSCT vaccination are recommended. Vaccination regimens for childhood immunization are clearly described in international guidelines, yet optimal timing of the vaccination is less clearly described and variable among guidelines [3,4,5,6,7].

Timing appears to play an important role in vaccine effectiveness. Vaccination before proper immune reconstitution may impair vaccine responses. However, as the risk of infection increases with time, postponing revaccination unnecessarily is undesirable [8,9]. Furthermore, recommendations for allogeneic HSCT (alloHSCT) and autologous HSCT (autoHSCT) recipients are uniform, whereas immunologic memory and immune reconstitution differ [3]. In daily practice, post-autoHSCT recipients are either not vaccinated or receive vaccination conforming with the post-alloHSCT guidelines. Insight into vaccine responses to childhood immunization vaccines related to timing of the vaccination is needed. The aim of the present review was to evaluate the current literature on optimal timing of vaccination, vaccine schedule and vaccine types post-HSCT in relation to vaccine responses.

## 2. Materials and Methods

### 2.1. Search Strategy

A PubMed search was conducted on 24 June 2021 using several terms, including MeSH terms, for the determinants ‘HSCT’ and ‘vaccination’ (Supplementary Table S1), combined with the Boolean operator ‘AND’. The search was limited to studies published after 1 January 2005. References of articles were crosschecked, and duplications were excluded.

### 2.2. Selection Criteria

Articles on children (age < 18), non-English written articles, reviews, case reports, recommendations (and similar: guidelines, opinions) and non-human studies (in-vitro and animal studies) were excluded. Full-text articles were selected by screening title and abstract for relevancy by the inclusion criteria: (1) study population being post-HSCT patients; (2) vaccinating the population post-HSCT; (3) measuring vaccine responses as correlates of protection post-vaccination.

### 2.3. Quality Assessment

Full-text articles were considered relevant when meeting inclusion criteria. All included articles were judged on their validity. For randomized controlled trials (RCT), the Cochrane Risk of Bias tool [10] was used, and for non-randomized cohort studies, the Robins-I Risk of Bias tool was used [11]. Two reviewers performed selection and quality assessment independently. Any disagreements were settled by consensus of the whole study group.

### 2.4. Data Extraction and Data Synthesis

To compare studies on both timing of vaccination and vaccine responses, data were extracted and tables were constructed per vaccine, displaying vaccine response as either seroprotection (meaning an antibody titer above vaccine-specific cutoff value) or seroconversion (conversion from a vaccine-specific negative antibody titer to a positive titer). Response rates after complete vaccine schedule are shown, unless otherwise indicated. Results on alloHSCT and autoHSCT are reported in separate paragraphs and each vaccine is discussed separately. Results are summarized in a table for both groups separately. Studies on both alloHSCT and autoHSCT recipients are reported in both paragraphs if results were available for separate subgroups of the study population; if only data on the total study population were reported, the study is reported in only one paragraph.

### 2.5. Post-Hoc Analysis

Due to rapidly evolving research on COVID-19 vaccines in HSCT patients and their relevance for these patients at this moment, an analysis of these studies was added after completion of the original study.

## 3. Results

### 3.1. Searching, Selection and Quality Assessment

**Figure 1:**
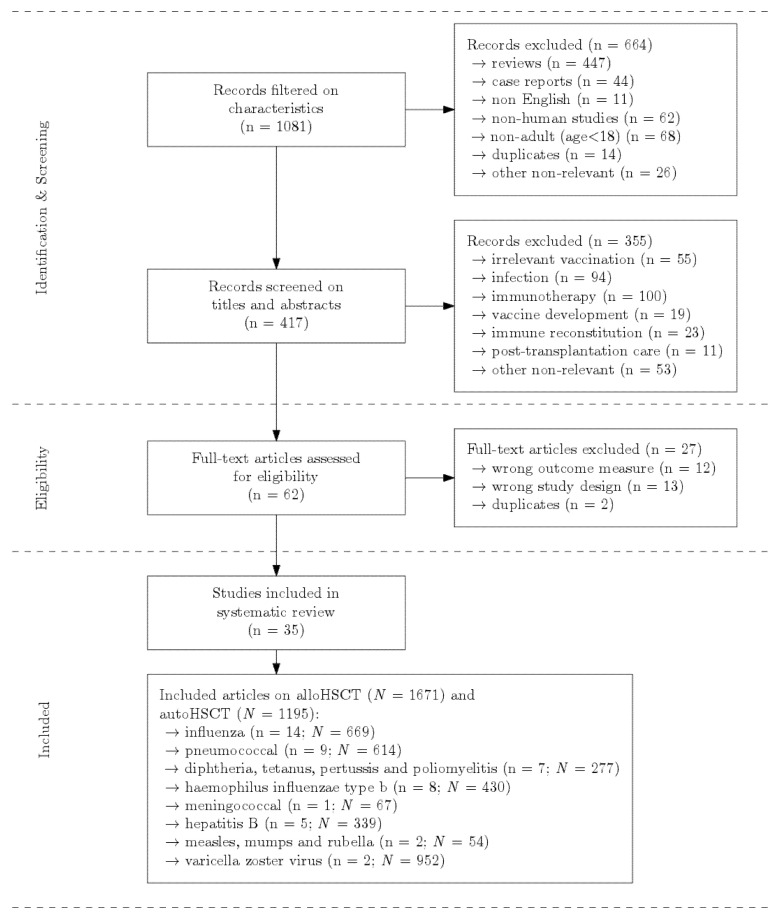
Flowchart of study selection. n = number of studies; *N* = number of patients in included studies.

The search generated 1081 articles (Figure 1). After exclusion for article characteristics, 417 articles were screened on title and abstract. Of these, 355 were excluded due to irrelevancy (for further detail see Figure 1). The selected 62 articles were assessed full-text, after which 27 were excluded due to irrelevant study design or wrong outcome measure or duplication. Consequently, 35 studies were included in the present comprehensive review, with a total of 2866 patients comprising 1671 alloHSCT recipients and 1195 autoHSCT recipients. A total of 24 articles reported results on alloHSCT, 5 on autoHSCT and 6 on both. Fourteen articles reported on influenza [12,13,14,15,16,17,18,19,20,21,22,23,24,25], four on pneumococci [26,27,28,29], one on tetanus and pertussis [30], one on Hib [31], one on meningococci [32], three on hepatitis B [33,34,35], two on measles, mumps and rubella (MMR) [36,37] and two on varicella zoster virus [38,39]. Seven articles reported data on multiple vaccines [40,41,42,43,44,45,46]. Study characteristics of included studies are shown in Supplementary Table S2. The quality assessment is shown in Supplementary Table S3, and definitions for vaccine responses as used in included studies are displayed in Supplementary Table S4.

### 3.2. Allogeneic Hematopoietic Stem Cell Transplantation

All results on alloHSCT patients are shown in Table 1.

#### 3.2.1. Influenza

The influenza vaccination in alloHSCT recipients has been widely studied: three RCT’s and eight cohort studies were identified. Response rates varied from 10% to 97%. First, administration of single influenza vaccine is discussed. Several studies reported that the timing of vaccination influenced the subsequent vaccine responses. However, an RCT was conducted comparing early single-dosage vaccination to late double-dosage vaccination, which did not show any differences in response for both timing and dosages [20]. Results from observational studies are more conflicting: Avetisyan [12] reported no differences in response between early-vaccinated and late-vaccinated patients, whereas others showed improved responses upon extending the delay of the administration of the influenza vaccination [13,19]. For example, Issa [13] reported improvement of response for every additional year of interval between transplantation and vaccination (OR 1.79 (1.12–2.85)). This was supported by De Lavallade [19], who reported that the patients vaccinated 48 months post-transplant gained seroprotection, whereas those who were vaccinated median 6.5 months post-transplant did not (*p* = 0.015). In addition to timing, usage of immunosuppressive medication has an influence on vaccine response: use of rituximab during the year prior to vaccination negatively influenced response [13]. Of note, post-HSCT patients were considered to have a delayed vaccine response compared with healthy controls: Mariotti measured 54% response at 28 days post-vaccination versus 71% response at 90 days post-vaccination [15].

Several studies compared different types of influenza vaccines. Natori [21] performed an RCT comparing the adjuvant vaccine and non-adjuvant vaccine; they found no difference. An RCT on high dose (HD) influenza vaccine as compared with standard dose (SD) showed no benefit of the HD vaccine [22].

Multiple studies administered two influenza vaccines. De Lavallade [19] reported improvement in response from 46% after the first vaccine to 73% after the second. Dhédin [16] and Mohty [18] supported this benefit of a second vaccine, but Karras [20] and Roll [17] did not. These differences might be explained by GVHD rates and timing. GVHD rates in the study by Roll [17] were much higher (36.8%) compared with the studies that demonstrated benefit of booster vaccination (De Lavallade 0% [19]; Mohty 15.4% [18]). Additionally, timing of vaccination was earlier in the study by Roll [17]: approximately 15 months versus 36 and 30 months, respectively [18,19], which may have influenced booster efficacy.

In conclusion, the data suggest that influenza responses in alloHSCT recipients is increased by postponing vaccination and that booster vaccination might be effective, whereas usage of adjuvants or high vaccine dosages does not improve vaccine responses.

#### 3.2.2. Pneumococcal

For pneumococcal vaccination in alloHSCT recipients, seven cohort studies and one RCT were included. There was huge variation in the number of vaccine dosages. Timing varied from 3 to 25 months post-transplant and responses varied from 43% to 99%.

To assess an optimal timing for vaccination, Cordonnier [29] performed an RCT to compare early (arm I: 3 months post-HSCT) to late (arm II: 9 months post-HSCT) vaccination using three conjugate vaccines (Prevenar PCV13 at 0, 1, 2 months) and one polysaccharide vaccine (Pneumovax PPV23 at 9 months). Responses were slightly better in arm II, which could possibly be explained by a higher rate of active GVHD in arm I (22.7% arm I versus 16.9% arm II). However, increase in response with increasing interval between HSCT and vaccination was supported by Langedijk [28]. Winkler [44], Meerveld-Eggink [41] and Shah [42] found comparable responses to Cordonnier [29] also using three conjugate vaccines (PCV7 or PCV13). Of note, Winkler [44] reported an improvement in measured vaccine response with time post-vaccination: measurement at 6 months post-vaccination showed seroconversion rates of 65–91% versus 55–67% at 1 month post-vaccination.

Usage of varying number of vaccines was studied. Usage of only one vaccine resulted in lower responses: 43–44.7% [26,40]. More recently, Cordonnier [27] evaluated immunogenicity of a fourth dose of PCV13 and concluded that this did not improve response. However, they did report better overall responses compared with their study in 2010. Some differences in study population might have played a role. The GVHD-rate was lower in 2015 (4.3% versus 16.9–22.7% in 2010) and conditioning included less myeloablative regimens (54.9% versus 79.6% in 2010).

Overall, best vaccine responses were obtained when three conjugate vaccines were administered. Vaccination initiated at 3 months post-HSCT provided adequate vaccine responses; however, postponing vaccination resulted in higher responses.

#### 3.2.3. Diphtheria, Tetanus, Pertussis (DTP) and Poliomyelitis

For diphtheria, tetanus, pertussis and poliomyelitis vaccination in alloHSCT recipients, four studies were included. Tetanus responses were 52–100% starting vaccination at least 6 months post-alloHSCT. Shah [42] and Meerveld-Eggink [41] reported better responses with respect to less acute GVHD and a prolonged interval between HSCT and vaccination. However, Conrad [43] and Winkler [44] showed higher response rates at 6–7.5 months post-transplantation, questioning the influence of interval between HSCT and vaccination. Winkler reported a worse seroconversion rate for polio, which is probably due to their already higher seroprotection rate pre-vaccination of 46% [44]. Conclusively, DTP and poliomyelitis vaccines might be administered at 6 months post-transplant.

#### 3.2.4. *Haemophilus influenzae* Type b (Hib)

Hib vaccination in alloHSCT recipients was evaluated in five cohort studies. All studies showed adequate responses of 77–97%. On short-term immunity, no benefit was found for administration of a booster vaccine [40,41,42,43,44]. Vaccination might be initiated at 6 months post-transplantation.

#### 3.2.5. Meningococcal

For meningococcal vaccination in alloHSCT recipients, two studies were identified, using meningococcal ACYW vaccine. Cheng [32] reported responses of 54–83% per serotype after one vaccine at 12 months post-HSCT.

#### 3.2.6. Hepatitis B

On hepatitis B vaccination in alloHSCT recipients, four studies were included. Onozawa [33] started vaccination at 12 months post-transplantation, and Takahata [34] started vaccination after cessation of immunosuppressive therapy—on average 15 months post alloHSCT. Both studies found comparable response rates around 40% after three vaccines. Jaffe [35] started their vaccination later—namely, 23 months post-transplant—and achieved better responses using the same vaccination schedule. Conrad [43] started earlier, at 6 months post-transplant and found a response rate of 84%. However, 56% of their population was already seroprotected before vaccination, which makes comparison more difficult. Of note, Jaffe [35] concluded that GVHD was associated with a significantly poorer response.

Conclusively, hepatitis B vaccination achieved poorer responses compared with all other vaccinations and should not be administered before 12 months, as responses seem to improve with time post-transplantation.

#### 3.2.7. Measles, Mumps and Rubella (MMR)

For MMR vaccination responses, two studies were included. Aoki [36] administered two doses of vaccine at median 69.3 months post-HSCT. All patients were seronegative before vaccination and seroconversion was found in only 19–30%. Evaluating their responses, chronic GVHD was negatively associated with seroconversion (OR 0.06; *p* = 0.023). This influence of GHVD is supported by Kawamura [37]. However, though vaccinating with only one dose and earlier post-transplantation (median 41.7 months post-HSCT), their seroconversion rates were remarkably better: 36–72%. This might partially be explained by different cutoff values for seroprotection.

#### 3.2.8. Varicella Zoster Virus (VZV)

For VZV vaccination, one study was included with Shingrix vaccine. This study only studied 17 alloHSCT patients [38]. Their response rate was 18%, which they compared with an autologous population (*n* = 13; response 62%). The only influencing factor in their multivariable analysis was type of HSCT, thus allogeneic or autologous (OR = 0.08; *p* = 0.01).

### 3.3. Autologous Hematopoietic Stem Cell Transplantation

All results on autoHSCT patients are shown in Table 2.

#### 3.3.1. Influenza

Data on influenza vaccination post-autoHSCT is scarce. Two studies reported only on autoHSCT recipients, of which one was an RCT by Villa [25]. This RCT compared single to double dosage vaccination at 1-year post-HSCT and reported no benefit from a second vaccine. Another study reported on autoHSCT and alloHSCT separately: Three autoHSCT recipients were included and vaccinated 2 years post-autoHSCT with response rates of 50–100% [14].

Gueller [23] and Engelhard [24] both reported on both autologous and allogeneic HSCT recipients. Respectively, 17.6% and 29.5% of the patients were autoHSCT recipients, with an average timing of vaccination of 20 months post-HSCT. Response rates were fairly low: 33–53% after one vaccine. Gueller reported benefit from booster vaccines improving response up to 91% [23], whereas Engelhard did not [24]. Assessing predictors for inadequate vaccine response, Gueller [23] concluded that usage of immunosuppressants caused failure in response: 88.9% of their non-responders used immunosuppressants versus 0% of their responders.

When comparing the autoHSCT population with the alloHSCT, some differences were reported. Yalçin [14] reported a better response in their autoHSCT recipients, and this is supported by Gueller [23]: they reported 71.9% of their seroconverters to be post-alloHSCT, whereas from their non-seroconverters 90% was post-alloHSCT.

#### 3.3.2. Pneumococcal

Only two studies on pneumococcal vaccination in autoHSCT recipients were included, with responses of 58–78% with vaccination started at 6–12 months post-HSCT [45,46]. Conclusively, the timing of this vaccination seems to make no difference and might therefore be initiated at 6 months post-autoHSCT.

#### 3.3.3. Diphtheria, Tetanus, Pertussis (DTP)

On diphtheria, tetanus and pertussis in autoHSCT patients, three studies were included. One study administering three dosages of DTP vaccine showed 100% response for tetanus, but all recipients appeared to already have protective antibody titers pre-vaccination [45]. The second study, also using three dosages, showed a seroconversion rate for tetanus of 60%, diphtheria 70% and pertussis 76%, with the majority of non-responders already being seroprotected pre-vaccination [46]. The final study vaccinated with a single DTP vaccine and reported low responses (7.1–32.1%). This study concluded that multiple vaccinations are required for adequate response [30]. Vaccination might thus be initiated at 6–12 months post-autoHSCT with preferably three vaccines to be administered.

#### 3.3.4. *Haemophilus influenzae* Type b (Hib)

For Hib vaccination in autoHSCT recipients, three studies were included. Both studies by Van der Velden showed high responses for vaccination at 6 months post-HSCT: 94–100% [31,45]. Palazzo found a lower seroconversion of 71%, yet 24% of their study population already had protective titers pre-vaccination [46].

#### 3.3.5. Meningococcal

Only one study on meningococcal vaccination in autoHSCT recipients was included. Cheng [32] reported comparable seroconversion rates for their alloHSCT and autoHSCT patients and suggested that, despite a response of 69–85% after one vaccine, a booster might be beneficial.

#### 3.3.6. Hepatitis B

On hepatitis B vaccination in autoHSCT patients, one study was included: Palazzo reported 40% seroconversion after three vaccinations [46].

#### 3.3.7. Varicella Zoster Virus (VZV)

For VZV vaccination, two studies were included with Shingrix vaccine. Both reported similar response rates, despite variety in moment of vaccination. Stadtmauer [39] included many more patients than Camargo [38] (*n* = 922 versus *n* = 13) and evaluated responses per age group with non-significant differences in response (age 18–49 years old, 57.7%; age > 49 years old, 71.4%). Conclusively, vaccination early post-autoHSCT might be feasible.

### 3.4. Overview of All Results

**Figure 2:**
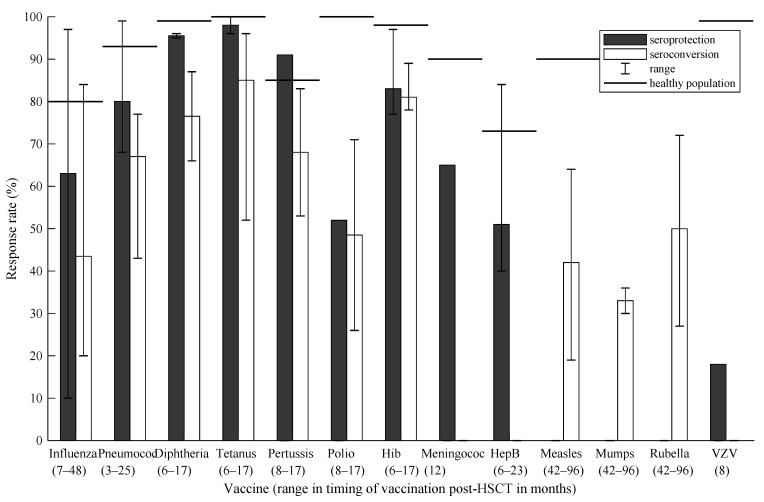
Response rates per vaccine in allogeneic HSCT recipients compared with the general healthy population. Polio = poliomyelitis; Hib = *Haemophilus influenzae* type b; HepB = hepatitis B; VZV = varicella zoster virus.

**Figure 3:**
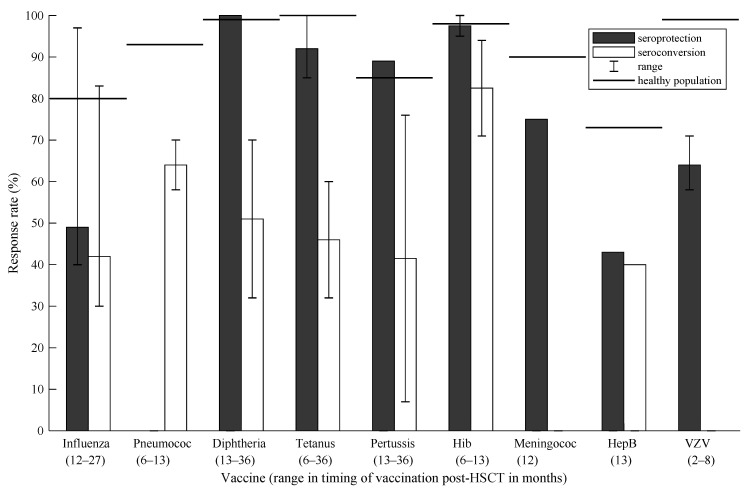
Response rates per vaccine in autologous HSCT recipients compared with the general healthy population. Abbreviations: Hib = *Haemophilus influenzae* type b; HepB = hepatitis B; VZV = varicella zoster virus.

In Figure 2 and Figure 3, responses per vaccine are visualized for alloHSCT and autoHSCT recipients in both seroprotection and seroconversion, respectively, and are compared with seroprotection rates post-vaccination in the general healthy population [47]. Timing is reported in months post-HSCT. In alloHSCT recipients, the influenza vaccine was administered at +7–48: seroprotection 10–97%; seroconversion 20–84%. Pneumococcal vaccination at +3–25: seroprotection 68–99%; seroconversion 43–77%. DTP, poliomyelitis and Hib at +6–17: seroprotection 77–100%; seroconversion 26–96%. Meningococcal vaccination at +12: seroprotection 65%. Hepatitis B at +6–23: seroprotection 40–84%. MMR vaccination at +41–69: seroconversion 19–72%. VZV vaccination at +8: 18%. For autoHSCT recipients, influenza at +12–27: seroprotection 40–97%; seroconversion 30–83. Pneumococcal vaccination at +6–13: seroconversion 58–70%. DTP and Hib at +6–36: seroprotection 85–100%; seroconversion 7–94%. Meningococcal and hepatitis B vaccination at +12 and +13, respectively: seroprotection at 75% and 43%, respectively. VZV at +2–8: 62–65%.

### 3.5. Post-Hoc Analysis: COVID-19

For studies on COVID-19 vaccines in post-HSCT patients, a separate search was conducted on 22 November 2021 using the original search strategy (Supplementary Table S1) with the addition of MeSH term ‘COVID-19’ or Title/abstract-term ‘covid*’ or ‘corona*’ through the Boolean operator ‘AND’. This search resulted in 77 results. These results were screened on title and abstract and were excluded based on similar criteria as studies in the original search. Namely, study design (case report *n* = 1; reviews *n* = 25; recommendations *n* = 4); not on HSCT patients (*n* = 21); not on vaccination (*n* = 9); not on COVID-19 (*n* = 3); wrong outcome measure (*n* = 4); duplication (*n* = 1). Only 1 article was included through snowballing. This resulted in 10 articles being suitable for this analysis, which are shown in Table 3 in order of timing of the vaccination post-HSCT [48,49,50,51,52,53,54,55,56,57].

Overall, all studies conclude worse response in case of early vaccination, especially when initiated before 12 months post-HSCT (OR = 5.72; *p* = 0.048) [50]. This was also seen in the available response rates for COVID vaccination: 37–50% <12 months post-HSCT [48,56] versus 68–94% >12 months post-HSCT [49,50,51,52,53,54,55,56]. One study reported on vaccination < 6 months post-HSCT with only 12.5% response (*n* = 8) [57]. In addition to timing, usage of immunosuppressants and active GVHD seem to be the most important factors influencing response. Conclusively, responses for COVID-19 vaccines remain poor compared with the healthy population long after HSCT, but responses do improve with time.

## 4. Discussion

This study intended to provide an overview of current knowledge on the optimal timing and schedule of post-HSCT vaccination. A total of 35 studies with varying vaccination schedules were included: 24 on alloHSCT, 5 on autoHSCT and 6 addressing both. In general, results are very heterogenic, and integrated studies are scarce.

### 4.1. Allogeneic Hematopoietic Stem Cell Transplantation

Drawing a firm conclusion based on the current literature is impossible due to the heterogeneity of the studies. Nevertheless, a few definitive points can be taken:

1. Many post-HSCT patients respond inadequately to vaccination, and responses are remarkably low compared with the healthy population. Thus, evaluating responses post-vaccination might be indicated. Suggested antibody titers for this evaluation in clinical practice are provided in Supplementary Table S5, including suggested cutoff values.
2. Most studies showed improved vaccine responses when delaying revaccination for at least 6 months post-transplantation and thereby allowing better immunological recovery [8,9]. However, postponing vaccination increases risk of infection: the estimated incidence of invasive pneumococcal disease (IPD) is 347 infections per 100,000 alloHSCT recipients compared with only 7 per 100,000 persons in the general population [28]. Timing of revaccination is a balance between immune recovery versus infection risk. Insights in individual immune recovery would help to assess the risk of failure. The factors that influence the immune system and the extent of influence are unknown.

For specific vaccines, some comments can be made in alloHSCT recipients:

1. Influenza vaccine studies were extremely heterogeneic. Responses seemed to improve with postponement of vaccination. Most studies initiated their vaccination at least 12 months post-transplantation, and therefore, no proper recommendation can be made on earlier vaccination. However, in case of a pandemic such as we experienced with COVID-19, a less adequate response to vaccination, and thus poorer seroprotection, would be preferable over no seroprotection [58]. Therefore, one might then consider vaccinating all patients that are at least 3 months post-HSCT and clinically stable. Earlier post-HSCT and vaccinating family members with close contact to the patient might be considered [4].
2. Pneumococcal vaccines might already be effective at 3–6 months post-transplant [27,29]. Earliest administration was three months post-transplant with response > 68%. However, responses did increase when vaccination was postponed: vaccination at 7–12 months post-transplant resulted in 69–99% responses [28,41,42,44].
3. Diphtheria, tetanus, pertussis, poliomyelitis and Hib were earliest administered at 6 months post-transplant. Responses for tetanus varied widely (52–100% [41,42,43,44]); for Hib responses were slightly more comparable between studies: 77–97% [40,44]. Vaccination on DTP, poliomyelitis and Hib might be started at 6 months post-transplantation.
4. Hepatitis B vaccine was administered 6–23 months post-HSCT with varying responses: 40–84% [33,34,35,43]. In the general population, responses to hepatitis B vaccine are also quite low compared with other vaccines [47,59]. Therefore, it is advised not to initiate vaccination before 12 months post-HSCT.
5. Vaccine responses to MMR remain suboptimal multiple years post-HSCT, and vaccination must be considered carefully per individual. As the vaccine is live-attenuated, the clinical condition of the individual patient is very important. Vaccination must be postponed in case of active GVHD and/or usage of immunosuppressants.
6. VZV vaccination has not yet been studied widely, and thus, no proper recommendation on timing can be made. However, with the recombinant subunit vaccine (Shingrix^®^), potential severe side effects of the live-attenuated VZV vaccine are no longer an issue, and vaccination can be considered earlier post-HSCT. Finally, COVID-19 vaccine responses are poor compared with the healthy population. However, vaccination should be considered shortly post-HSCT, as any chance for response must be taken in times of a pandemic such as COVID-19.

### 4.2. Autologous Hematopoietic Stem Cell Transplantation

For vaccination of autologous HSCT patients, fewer data are available. Although guidelines [3,4,5,6,7] recommend vaccination post-autoHSCT, data on vaccinating this population are scarce. One can postulate that responses post-autoHSCT might be better because of maintenance of pre-transplant-obtained immunity in post-autoHSCT recipients [60]. For post-autoHSCT patients, revaccination is not always general practice despite it being recommended in current guidelines, similar to guidelines for post-alloHSCT patients. Data on vaccination of post-autoHSCT patients are scarce; however, responses seem to be slightly better in this group compared with post-alloHSCT patients. A more tailored approach might therefore be indicated. However, one could question whether that would be cost-effective.

An important factor to consider for this more individual approach in autoHSCT patients would be the original indication of the transplantation. Additional immunosuppressive treatment post-autoHSCT might decrease vaccine responses [61]. For example, rituximab maintenance in mantle cell lymphoma likely has a significant suppressive effect due to continuous B cell depletion. Likewise, lenalidomide maintenance post-autoHSCT for multiple myeloma patients could have an impact on vaccine responses. On the one hand lenalidomide can induce leukopenia, but it is also associated with immune modularity properties resulting, for example, in increased incidence of GVHD [62]. The current evidence of Palazzo et al. [46] does however not suggest a significant immunosuppressive effect of lenalidomide on vaccine response. Any further supporting evidence with respect to live attenuated vaccines in the setting of lenalidomide would be helpful. Of note, some guidelines still suggest avoiding these live vaccines whilst on lenalidomide maintenance post-autoHSCT beyond two years. However, interestingly, recommendations for non-autoHSCT patients on similar medication are lacking. Nevertheless, we recommend being cautious with live attenuated vaccines in patients with multiple myeloma because not only medication but also the disease itself as well as aging contribute to the risk of vaccine-related disease.

### 4.3. Overall Discussion

Some general recommendations can be made:

1. Physicians may need to take into account the current state of immunosuppression and associated expectations for immune recovery to determine the precise timing and the estimated success of vaccination post-HSCT.
2. Future studies with standardized definitions, especially on vaccine response (seroconversion/seroprotection), are necessary to increase generalizability and relevancy of results.
3. More insight in predictors of vaccine response, such as immune reconstitution, GVHD-status and immunosuppressive therapy [61], might help to design an optimized and probably more individualized vaccination schedule. For example, rituximab is known to interfere with immune recovery for 6–12 months after cessation [63] and was reported to negatively influence vaccine response [13]. Therefore, postponing vaccination is necessary in these specific patients.
4. Finally, though evaluating vaccine responses post-vaccination is highly recommended, it is important to note that antibody titers might still increase with time due to possibly delayed immunization in the post-HSCT population [15,44].

The literature search was performed extensively and considered to be as complete as possible. Accuracy was assured by studying each vaccine separately and also by displaying both seroprotection and seroconversion rates to allow optimal comparison among data. To ensure the quality of this study, great effort was made to maintain proper validity and reliability throughout all included studies. However, there were some limitations. First, only antibody titers were evaluated as a measure of vaccine response, which might underestimate vaccine effectiveness: patients might still benefit from vaccination despite low antibody responses [64]. Furthermore, only the effect of the first vaccination post-HSCT was evaluated, and no conclusion can therefore be drawn on effectiveness of booster vaccination. Finally, as only childhood immunizations were studied, additional vaccines might be indicated regionally, such as the hepatitis A or yellow fever vaccine.

## 5. Conclusions and Recommendations

In conclusion, vaccine responses to the childhood immunization vaccines in post-HSCT patients are lower as compared with healthy individuals, and therefore, measurement of response might be indicated. Furthermore, timing of vaccination is essential to reach optimal responses. Considering the influence of patient factors such as GVHD and usage of immunosuppressive drugs, an individualized approach might be necessary to optimize vaccine responses. However, guidance on how to initiate this individualized approach is currently lacking and further studies are needed.

Based on the included studies, general recommendations on timing of the childhood immunization vaccines post-alloHSCT are made and shown in Table 4. Due to overall lower response rates compared with healthy populations [47], assessment of response after vaccination is recommended. For autoHSCT recipients, no recommendations could be formed based on our review due to the scarcity of data.

## Figures and Tables

**Table 1:** Results on vaccination in allogeneic HSCT recipients.

	Study	*N*	Vaccine Schedule		Response Rate (%) *	
			Start of Vaccination, in Months (Range)	Schedule in Days	Seroprotection	Seroconversion
Influenza	Avetisyan et al. [12]	14	+9.2 (3–24)	0	29%, 0%, 0% **	
	Issa et al. [13]	82	+19 (2.5–94)	0	51%	
	Yalçin et al. [14]	58	+24.2 (6–48)	0	91% ***, 100%, 100% **	59.1%, 54.5%, 47.7% **
	Mariotti et al. [15]	15	+48 (7–116)	0	71.4%	57.1%
	Dhédin et al. [16]	59	+11 (5.1–24.8)	0, 21	After 1st 53% After 2nd 66%	After 1st 51% After 2nd 66%
	Roll et al. [17]	38	+15 (5.1–96.4)	0, 35 (range 14–70)	After 1st 42% After 2nd 47%	After 1st 42%
	Mohty et al. [18]	57	+30 (2–192)	0, 21	After 1st 64.3% After 2nd 84.2%	After 1st 53.8% After 2nd 84.2%
	De Lavallade et al. [19]	26	+36 (6–127)	0, 21	After 1st 46% After 2nd 73%	After 1st 46% After 2nd 73%
	Karras et al. [20] ****	65	Arm I: +8.4 (2.4–236.4) Arm II: +12 (2.4–84)	Arm I: 0 Arm II: 0, 30	Arm I: 32%, 19%, 32% ** Arm II: 32%, 19%, 23% **	Arm I: 31% 13% 16% ** Arm II: 31% 22% 25% **
	Natori et al. [21]	73	+12.6 (2.8–270)	Arm I: 0 (adjuvant vaccine) Arm II: 0 (non-adjuvant vaccine)	Arm I: 57.1%, 71.4%, 57.1% ** Arm II: 59.4%, 71.9%, 68.8% **	Arm I: 31.4%, 57.1%, 37.1% ** Arm II: 21.9%, 40.6%, 25.0% **
	Halasa et al. [22]	44	Arm I: +8.5 Arm II: +7.1	Arm I: 0 (60 µg) Arm II: 0 (15 µg)	Arm I: 69%, 81%, 42% ** Arm II: 57%, 36%, 43% **	Arm I: 50%, 42%, 42% ** Arm II: 36%, 36%, 43% **
Pneumococcal	Pao et al. [40]	76	+15.6	0 (PCV7)		44.7%
	Okinaka et al. [26]	30	+25.2 (13.0–63.4)	0 (PPV23)		43%
	Cordonnier et al. [27]	162	+4.9 (3.2–6.8)	0, 1, 2, 8 (PCV13), 9 (PPV23)	After 3× PCV13 89.7–98%, after 4× PCV13 82.6–98.8% *****	
	Winkler et al. [44]	27	+7.5 (6.0–14.3)	0, 1, 2 (PCV13)	Serotype 1 96%, 14 100%, 23 100%	Serotype 1 74%, 14 65%, 23 91%
	Meerveld-Eggink et al. [41]	26	+15 (12–36)	0, 0.5, 2 (PCV7)	Serotype 4 73%, 6B 28% 9V 78%, 14 86%, 18C 82%, 19F 73%, 23F 82%	Serotype 4 85%, 6B 46%, 9V 73%, 14 69%, 18C 69%, 19F 69%, 23F 62%
	Shah et al. [42]	63	+17 (7–45)	0, 1, 2 (PCV7/PCV13)		Serotype 14 65%, 19F 77%, 23F 58%
	Langedijk et al. [28]	103	+20.3 (2.2–164.4)	0, 1, 2 (PCV13), 6 (PPV23)	PCV13 serotypes 85%, PPV23 serotypes 23–92%	
	Cordonnier et al. [29] ******	158	Arm I: +3 Arm II: +9	Arm I: 0, 1, 2, PCV13, 9 (PPV23) Arm II: 0, 1, 2 (PCV13), 9 (PPV23)	Arm I before PPV23 59%, after PPV23 68% Arm II before PPV23 69%, after PPV23 88%	
DTP and poliomyelitis	Conrad et al. [43]	91	+6	0, 1, 2	Diphtheria 95%, tetanus 98%	
	Winkler et al. [44]	27	+7.5 (6.0–14.3)	0, 1, 2	Diphtheria 96%, tetanus 100%, pertussis 91%, poliomyelitis 52%	Diphtheria 87%, tetanus 96%, pertussis 83%, poliomyelitis 26%
	Meerveld-Eggink et al. [41]	26	+15 (12–36) DTP	0, 2	Tetanus 96%	Tetanus 85%
	Shah et al. [42]	63	+17 (7–45) DTP +17 (7–45) poliomyelitis	0, 1, 2 DTP; 0, 1 poliomyelitis		Diphtheria 66%, tetanus 52%, pertussis 53%, poliomyelitis 71%
Hib	Conrad et al. [43]	91	+6	0, 1, 2	97%	
	Winkler et al. [44]	27	+7.5 (6.0–14.3)	0, 1, 2	83%	83%
	Meerveld-Eggink et al. [41]	26	+15 (12–36)	0	77%	78%
	Pao et al. [40]	65	+15.6	0		79%
	Shah et al. [42]	63	+17 (7–45)	0, 1, 2		89%
Men ACYW	Cheng et al. [32]	54	+12.3 (8.4–13.5)	0	Serotype A 83.3%, C 61.1%, Y 63.0%, W-135 53.7%	
Hepatitis B	Conrad et al. [43]	15	+6	0, 1, 2	84%	
	Onozawa et al. [33]	13	+12	0, 1, 6	40%	
	Takahata et al. [34]	21	+15 (6–79)	0, 1, 6	42.9%	
	Jaffe et al. [35]	168	+23 (5–102)	0, 1, 6	59%	
MMR	Kawamura et al. [37]	25	+41.7 (24.4–99.1)	0		Measles 64%, mumps 36%, rubella 72%
	Aoki et al. [36]	29	+69.3 (25.8–212.6)	0, 1		Measles 19%, mumps 30%, rubella 27% *******
VZV	Camargo et al. [38]	17	+8 (7–12)	0, 2	18%	

Start of vaccination is displayed in relation to moment of HSCT. *N* = number of vaccinated post-HSCT patients in the study population. * Response rates are for serotype A/H1N1 unless otherwise indicated. ** Respectively, serotypes A/H1N1, A/H3N2, B. *** 2 autologous stem cell recipients included in this percentage. **** 26.2% of study population < 18 years old. ***** Percentage is based on the whole study population consisting of 25% of patients < 18 years old. ****** 9.5% < 16 years old divided equally among both study arms; 101/158 patients received PPV23 vaccine. ******* 8 autologous stem cell recipients included in these percentages.

**Table 2:** Results on vaccination in autologous HSCT recipients.

	Study	*N*	Vaccination Schedule and Timing		Response Rate (%) *	
			Start of Vaccination, in Months (Range)	Schedule, in Weeks	Seroprotection	Seroconversion
Influenza	Gueller et al. [23]	17 **	+19.7 (4.7–49.3)	0, 3	After 1st 52.9% After 2nd 90.9%	After 1st 41.2% After 2nd 81.8%
	Yalçin et al. [14]	3	+24.2 (6–48)		91% ***, 100%, 100% ****	100%, 100%, 50% ****
	Engelhard et al. [24]	78 *	+27 (1–290) *****	0, 3–4	After 1st 44.2% After 2nd 48.8%	After 1st 32.5% After 2nd 41.9%
	Villa et al. [25]	40	Arm I: +12 months Arm II: +12 months	Arm I: 0 Arm II: 0, 3	Arm I: 40% Arm II: after 1st 15%, after 2nd 40%	Arm I: 30% Arm II: after 1st 5%, after 2nd 30%
Pneumococcal	Van der Velden et al. [45]	20	+6	0, 2 (PCV7), 8 (PPV23)	PCV7 serotypes 78%, PPV23 serotypes 61%	
	Palazzo et al. [46]	122	+12.6 (8.1–26.4)	0, 1, 2 (PCV13)	58%	
DTP	Van der Velden et al. [45]	20	+6	0, 2, 8	Tetanus 100%	
	Palazzo et al. [44]	122	+12.6 (8.1–26.4)	0, 1, 2	Tetanus 92%, diphtheria 100%, pertussis 89%	Tetanus 60%, diphtheria 70%, pertussis 76%
	Small et al. [30]	28	+36 (15.6–99.9)	0	Tetanus: 85% *	Tetanus 32.1%, diphtheria 32.1, pertussis 7.1%
Hib	Van der Velden et al. [31]	16	+6	0, 2, 8	100%	
	Van der Velden et al. [45]	20	+6	0, 2, 8		After 1st 33% After 2nd 72% After 3rd 94%
	Palazzo et al. [46]	122	+12.6 (8.1–26.4)	0, 1, 2	95%	71%
Men ACYW	Cheng et al. [32]	13 *	+12.3 (8.4–13.5)		Serotype A 69.2%, C 84.6%, Y 76.9%, W-135 69.2%	
HepB	Palazzo et al. [46]	122	+12.6 (8.1–26.4)	0, 1, 6	43%	40%
VZV	Stadtmauer et al. [39]	922	+2	0, 1–2	57.7–71.4%	
	Camargo et al. [38]	13	+8 (7–12)	0, 2	62%	

Start of vaccination is displayed in relation to moment of HSCT. *N* = number of vaccinated post-HSCT patients in the study population. * Response rates are for serotype A/H1N1 unless otherwise indicated. ** Autologous patients included: 17.6% [23], 29.5% [24]; results are on total study population. *** Only two autologous stem cell recipients included in this percentage. **** Respectively serotypes A/H1N1, AH3N2, B. ***** Allogeneic recipients median 34 months post-transplant, autologous recipients median 20 months post-transplant.

**Table 3:** Results on COVID-19 vaccination in HSCT recipients.

Study	*N*	Allo/Auto	Start of Vaccination, in Months (Range)	Vaccine	Schedule, in Weeks	Seroconversion
Chiarucci et al. [48]	50	12 allo + 38 auto	12.3 (0.2–24.5)	Pfizer-BioNTech	0, 3	Allo: 50% Auto: 84%
Lindemann et al. [49]	117	Allo	30 (5–391)	Pfizer-BioNTech	0, 3	68%
Canti et al. [50]	37	Allo	31 (5–51)	Pfizer-BioNTech	0, 3	86%
Ram et al. [51]	66	Allo	32 (3–263)	Pfizer-BioNTech	0, 3	75%
Tamari et al. [52]	217	149 allo + 61 auto	34 (16–59)	Pfizer-BioNTech/Moderna	0, 3	87%
Shem-Tov et al. [53]	152	Allo	+41 (24–76)	Pfizer-BioNTech	0, 3	77.6%
Luis Pinana et al. [54]	397	311 allo + 86 auto	Allo: 98 (4–646) Auto: 88 (3–763)	Pfizer-BioNTech/ AstraZeneca/Moderna	0, 3	Allo: 78% Auto: 85%
Matkowska-Kocjan et al. [55]	65	Allo	126 (36–324)	Pfizer-BioNTech	0, 5	96.5%
Attolico et al. [56]	114	52 allo + 52 auto	Non available *	Pfizer-BioNTech	0,3	Allo: 75.8% Auto: 94.2%
Easdale et al. [57]	55	Allo	Non available **	Pfizer-BioNTech/AstraZeneca	0	38.2%

* Patients were divided into three groups: G1: vaccination < 1 year post-HSCT (*n* = 19); G2: 1–5 years post-HSCT (*n* = 52); and G3: >5 years post-HSCT (*n* = 43). Responses: G1 37%; G2 94%; G3 93%. ** Patients were divided into two groups: G1: vaccination 3–6 months post-HSCT (*n* = 8); G2: >6 months post-HSCT. Responses: G1 12.5%; G2 42.6%.

**Table 4:** Recommendations on timing of vaccination in allogeneic HSCT recipients *.

Vaccine	Number of Vaccines	Schedule	
		First Vaccination, in Months Post-HSCT	Schedule, in Months
Influenza	1	12 months **	Yearly in influenza season
Pneumococcal (PCV13)	3× PCV13, 1× PPV23	PCV13: 3–6 months ***	PCV13: 0, 1, 2 months PPV23: 9 months
Diphtheria, tetanus, pertussis and poliomyelitis	3	6 months	0, 1, 2 months
*Haemophilus influenzae* type b	3	6 months	0, 1, 2 months
Hepatitis B	3	12 months	0, 1, 6 months
Meningococcal	2	12 months	0, 2–8 months
Measles, mumps and rubella	1	24 months ****	0
Varicella zoster virus (Shingrix^®^)	2	12 months	0, 1 months

* Consider postponing vaccination when rituximab was used 6–12 months prior to scheduled vaccination. ** Vaccination might be antedated for pragmatic reasons (flu season) up to as early as 3 months post-HSCT. *** Consider postponing vaccination for patients with myeloablative conditioning or acute GVHD. **** Only in the case of no active GVHD and no active usage of immunosuppressants.

## Supplementary Materials

The following are available online at https://www.mdpi.com/article/10.3390/cancers13236140/s1, Table S1: Search strategy in PubMed; Table S2: Study characteristics and validity of included studies; Table S3A: Cochrane Risk of Bias tool for Randomized Controlled Trials*; Table S3B: Robins-I Risk of Bias tool for Non-randomized Studies of Interventions*; Table S3C: Legend for supplement table 3A (Cochrane Risk of Bias tool for Randomized Controlled Trials); Table S3D: Legend for supplement table 3B (Robins-I Risk of Bias tool for Non-randomized Studies of Interventions); Table S4: Used definitions for response vaccine response per included study; Table S5: Suggestions for evaluation of vaccine response in clinical practice.
