# Supplementary material for: Vaccine Responses in Adult Hematopoietic Stem Cell Transplant Recipients: A Comprehensive Review
Source: Cancers (Basel). 2021 Dec 6;13(23):6140. doi: 10.3390/cancers13236140 (PMC8656479; doi:10.3390/cancers13236140)
Supplement: Supplementary file 1 [file cancers-13-06140-s001.zip › cancers-1469563-supplementary.pdf]

cancers

MDPI

**Table S1.** Search strategy in PubMed.

| | Determinant 1 | Determinant 2 |
|---|---|---|
| Title/abstract | Stem cell transplant<br>HSCT<br>SCT | Vaccin |
| Mesh-term | Stem cell transplantation | Vaccination |

**Table S2.** Study characteristics and validity of included studies.

| Reference | Year | Country | *N* | HSCT |
|---|---|---|---|---|
| Van der Velden et al[31] | 2005 | Netherlands | 16 | Auto |
| Jaffe et al[35] | 2006 | United States | 168 | Allo |
| Van der Velden et al[45] | 2007 | Netherlands | 20 | Auto |
| Avetisyan et al[12] | 2008 | Sweden | 14 | Allo |
| Pao et al[40] | 2008 | United States | 76 | Allo |
| Onozawa et al[33] | 2008 | Japan | 13 | Allo |
| Meerveld-Eggink et al[41] | 2009 | Netherlands | 26 | Allo |
| Small et al[30] | 2009 | United States | 28 | Auto |
| Cordonnier et al[29] | 2009 | France | 158 | Allo |
| Yalçin et al[14] | 2010 | Turkey | 61 | Allo+auto |
| Issa et al[13] | 2011 | United States | 82 | Allo |
| De Lavallade et al[19] | 2011 | United Kingdom | 26 | Allo |
| Mohty et al[18] | 2011 | Switzerland | 57 | Allo |
| Gueller et al[23] | 2011 | Germany | 17 | Allo+auto |
| Engelhard et al[24] | 2011 | Israel | 78 | Allo+auto |
| Roll et al[17] | 2012 | Germany | 38 | Allo |
| Mariotti et al[15] | 2012 | Italy | 15 | Allo |
| Villa et al[25] | 2012 | Canada | 40 | Auto |
| Karras et al[20] | 2013 | United States | 65 | Allo |
| Dhédin et al[16] | 2014 | France | 59 | Allo |
| Takahata et al[34] | 2014 | Japan | 21 | Allo |
| Shah et al[42] | 2015 | United States | 63 | Allo |
| Cordonnier et al[27] | 2015 | France | 162 | Allo |
| Natori et al[21] | 2016 | Canada | 73 | Allo |
| Halasa et al[22] | 2016 | United States | 44 | Allo |
| Okinaka et al[26] | 2017 | Japan | 30 | Allo |
| Palazzo et al[46] | 2018 | United States | 122 | Auto |
| Cheng et al[32] | 2018 | United States | 67 | Allo+auto |
| Langedijk et al[28] | 2019 | Netherlands | 103 | Allo |
| Aoki et al[36] | 2019 | Japan | 29 | Allo |
| Conrad et al[43] | 2020 | France | 91 | Allo |
| Winkler et al[44] | 2020 | Germany | 27 | Allo |
| Camargo et al[38] | 2020 | United States | 30 | Allo+auto |
| Stadtmauer et al[39] | 2021 | United States | 922 | Auto |
| Kawamura et al[37] | 2021 | Japan | 25 | Allo |

*N* = number of vaccinated post-HSCT patients in study population; auto = autologous HSCT recipients; allo = allogeneic HSCT recipients.

**Table S3A.** Cochrane Risk of Bias tool for Randomized Controlled Trials*.

| Study | Randomization process | Deviation from intended intervention | Missing outcome data | Measurement of outcome | Selection of reported result |
|---|---|---|---|---|---|
| Cordonnier et al[29] | Low | Low | Low | Low | Low |
| Villa et al[25] | Low | Low | Low | Low | Low |
| Karras et al[20] | Low | Low | Low | Low | Low |
| Natori et al[21] | Low | Low | Low | Low | Low |
| Halasa et al[22] | Low | Low | Moderate | Low | Low |
| Stadtmauer et al[39] | Low | Low | Low | Low | Low |

● = low risk; ● = moderate risk; ● = serious risk; *The legend is displayed in supplement table 3C.

**Table S3B.** Robins-I Risk of Bias tool for Non-randomized Studies of Interventions*.

| Study | Confounding | Selection | Intervention classification | Deviation from intended intervention | Missing data | Measurement of outcome | Selection of reported result |
|---|---|---|---|---|---|---|---|
| Van der Velden et al[31] | Low | Moderate | Low | Moderate | Low | Low | Low |
| Jaffe et al[35] | Low | Low | Low | Low | Moderate | Low | Low |
| Van der Velden et al[45] | Low | Moderate | Low | Moderate | Moderate | Low | Low |
| Avetisyan et al[12] | Low | Serious | Low | Low | Moderate | Low | Low |
| Pao et al[40] | Low | Moderate | Low | Low | Low | Low | Low |
| Onozawa et al[33] | Low | Moderate | Low | Low | Low | Low | Low |
| Meerveld-Eggink et al[41] | Low | Moderate | Low | Low | Moderate | Low | Low |
| Small et al[30] | Low | Moderate | Low | Moderate | Moderate | Low | Low |
| Yalçin et al[14] | Low | Moderate | Low | Low | Moderate | Low | Low |
| Issa et al[13] | Serious | Moderate | Low | Low | Low | Low | Low |
| De Lavallade et al[19] | Low | Low | Low | Low | Moderate | Low | Low |
| Mohty et al[18] | Low | Low | Low | Low | Low | Low | Low |
| Gueller et al[23] | Low | Moderate | Low | Low | Serious | Low | Low |
| Engelhard et al[24] | Low | Moderate | Low | Low | Moderate | Low | Low |
| Roll et al[17] | Low | Moderate | Low | Moderate | Moderate | Low | Low |
| Mariotti et al[15] | Low | Low | Low | Low | Moderate | Low | Low |
| Dhédin et al[16] | Low | Moderate | Low | Low | Low | Low | Low |
| Takahata et al[34] | Moderate | Moderate | Low | Low | Low | Low | Low |
| Shah et al[42] | Low | Low | Low | Low | Low | Low | Low |
| Cordonnier et al[27] | Low | Low | Low | Low | Low | Low | Low |
| Okinaka et al[26] | Low | Moderate | Low | Low | Low | Low | Low |
| Palazzo et al[46] | Low | Moderate | Low | Low | Low | Low | Low |
| Cheng et al[32] | Low | Moderate | Low | Low | Low | Low | Low |
| Langedijk et al[28] | Serious | Low | Low | Low | Low | Low | Low |
| Aoki et al[36] | Low | Low | Low | Low | Low | Low | Low |
| Conrad et al[43] | Low | Low | Low | Moderate | Low | Low | Low |
| Winkler et al[44] | Low | Moderate | Low | Low | Moderate | Low | Low |
| Kawamura et al[37] | Low | Low | Low | Low | Low | Low | Low |
| Camargo et al[38] | Low | Low | Low | Low | Serious | Low | Low |

● = low risk; ● = moderate risk; ● = serious risk; *The legend is displayed in supplement table 3D.

**Table S3C.** Legend for supplement table 3A (Cochrane Risk of Bias tool for Randomized Controlled Trials).

| | |
|---|---|
| Randomization process | ● allocation random and concealment guaranteed<br>● allocation concealment not precisely described or not adequately guaranteed<br>● allocation not random or no concealment guaranteed |
| Deviation from intended intervention | ● complete population vaccinated according to pre-set schedule or slight deviation from set schedule reported adequately without disturbance of the balance between groups<br>● no accurate data on actual moment of vaccination but considered not to be concerning for the outcomes and without disturbance of the balance between groups<br>● deviation from pre-set schedule that is concerning for the outcomes or with disturbance of the balance between groups |
| Missing outcome data | ● no missing data or missing data with properly reported reasoning<br>● missing data without reported reasoning but not concerning for outcomes and balance<br>● missing data that is concerning to influence the outcomes or to disturb balance |
| Measurement of outcome | ● methods clearly described and considered adequate and were equal between groups<br>● methods not clearly described but considered adequate and were equal between groups<br>● methods considered inadequate or were inequal between groups |
| Selection of reported result | ● complete and extensive report of all results according to pre-specified analysis plan<br>● reporting of results complete but not (properly) extensive<br>● incomplete reporting of results or not in accordance with pre-specified analysis plan |

**Table S3D.** Legend for supplement table 3B (Robins-I Risk of Bias tool for Non-randomized Studies of Interventions).

| | |
|---|---|
| Confounding | ● pre-vaccination titers available and taken into analysis<br>● no pre-vaccination titers available but data available on previous vaccination or immune status<br>● no pre-vaccination titers and no data on previous vaccination or immune status available |
| Selection | ● study population representative for target population<br>● possible selection of patient groups but not-concerning<br>● selection of patient groups that is concerning for the outcomes |
| Intervention classification | ● clearly pre-set schedule for vaccinating study population<br>● schedule for vaccinating study population not clearly set<br>● no pre-set schedule for vaccinating study population |
| Deviation from intended intervention | ● whole study population vaccinated according to the pre-set schedule or deviation from set schedule with adequate reporting<br>● no accurate data on actual moment of vaccination but considered not to be concerning for the outcomes<br>● deviation that is possibly concerning for the outcomes |
| Missing data | ● no missing data or missing data with properly reported reasoning<br>● missing data without reported reasoning but not concerning for outcomes<br>● missing data that is concerning to influence the outcomes |

| | |
|---|---|
| Measurement of outcome | ● methods used for outcome clearly described and considered adequate<br>● methods used for outcome not clearly described but considered adequate<br>● methods used for outcome considered inadequate |
| Selection of reported results | ● complete and extensive report of all results according to pre-specified analysis plan<br>● reporting of results complete but not (properly) extensive<br>● incomplete reporting of results or not in accordance with pre-specified analysis plan |

**Table S4.** Used definitions for response vaccine response per included study.

| Vaccine | Definition for response | | Studies using this definition |
|---|---|---|---|
| | Seroprotection | Seroconversion | |
| **Influenza** | Absolute titer >1:40 | Pre-vaccination titer ≤ 1:10 and post-vaccination ≥ 1:40 or pre-vaccination > 1:10 and post-vaccination ≥4-fold rise in titer | Villa et al,[25] Gueller et al,[23] Engelhard et al,[24] Roll et al,[17] Mariotti et al,[15] Dhédin et al,[16] Mohty et al[18] |
| | Absolute titer ≥1:40 | Post-vaccination ≥4-fold rise in titer | Karras et al,[20] Natori et al,[21] Halasa et al,[22] Yalçin et al[14] |
| | Absolute titer ≥1:40 | | Avetisyan et al,[12] Issa et al[13] |
| | Absolute titer ≥1:32 | Pre-vaccination titer <1:8 and post-vaccination ≥1:32 or pre-vaccination ≥1:8 and post-vaccination ≥4-fold rise in titer | De Lavallade et al[19] |
| **Pneumococcus** | | ≥2-fold rise in titer and absolute titer ≥0.35 ug/ml for ≥2/3 tested serotypes | Van der Velden et al[45] |
| | Absolute titer ≥0.35 µg/ml | ≥4-fold rise in titer | Meerveld-Eggink et al,[41] Winkler et al[44] |
| | Absolute titer >0.15ug/ml for all PCV7 serotypes | | Cordonnier et al[29] |
| | Absolute titer ≥0.35 µg/ml | | Cordonnier et al,[27] Langedijk et al[28] |
| | | ≥3-fold rise in titer for serotypes 14,1 9f and 23f | Pao et al,[40] Shah et al[42] |
| | | ≥2-fold rise in titer | Okinaka et al[26] |
| | | ≥2-fold rise in titer for ≥70% of serotypes | Palazzo et al[46] |
| **Diphtheria** | | ≥4-fold rise in titer | Small et al[30] |
| | Absolute titer ≥0.01 IU/ml | ≥4-fold rise in titer | Palazzo et al[46] |
| | | ≥3-fold rise in titer | Shah et al[42] |
| | Absolute titer ≥0.1 IU/ml | ≥4-fold rise in titer | Winkler et al[44] |
| | Absolute titer ≥0.1 IU/ml | | Conrad et al[43] |
| **Tetanus** | Absolute titer ≥0.1 IU/ml | ≥4-fold rise in titer | Meerveld-Eggink et al,[41] Winkler et al[44] |
| | Absolute titer ≥0.15 IU/ml | ≥4-fold rise in titer | Palazzo et al[46] |
| | | ≥4-fold rise in titer | Small et al[30] |
| | | ≥3-fold rise in titer | Shah et al[40] |
| | Absolute titer ≥0.1 IU/ml | | Conrad et al[43] |
| | Absolute titer 0.01 IU/ml | | Van der Velden et al[45] |
| **Pertussis** | | ≥2-fold rise in titer | Small et al,[30] Shah et al[42] |
| | Absolute titer ≥ 24 IU/ml | ≥4-fold rise in titer | Winkler et al[44] |
| | Absolute titer >5 IU/ml | Titer increase to >5 IU/ml | Palazzo et al[46] |
| **Poliomyelitis** | Absolute titer ≥ 10 U/ml | ≥4-fold rise in titer and ≥ 100% increase between pre-vaccination and post-vaccination | Winkler et al[44] |
| | | ≥3-fold rise in titer | Shah et al[42] |
| ***Haemophilus influenzae* type b** | Absolute titer ≥1.0 ug/ml | | Conrad et al,[43] Van der Velden et al[31] |
| | Absolute titer ≥1.0 ug/ml | ≥4-fold rise in titer and ≥ 100% increase between pre-vaccination and post-vaccination | Winkler et al[44] |

| | | | |
|---|---|---|---|
| | | ≥4-fold rise in titer and post-vaccination levels ≥6.16 ug/ml | Van der Velden et al[45] |
| | Absolute titer ≥1.0 ug/ml | Conversion from non-protective (<0.15 ug/ml) to protective (≥1.0 ug/ml) or ≥4-fold rise in titer if in indeterminate range | Palazzo et al[46] |
| | | ≥3-fold rise in titer | Shah et al[42] |
| | | Conversion to >1 ug/ml or >3-fold increase titer | Pao et al[40] |
| | | ≥4-fold rise in titer | Meerveld-Eggink et al[41] |
| **Meningococcus** | **Serum Bacterial Activity (SBA) titers ≥1:8** | | Cheng et al[32] |
| **Hepatitis B** | Seroprotection anti-Hbs >10 mIU/ml | | Onozawa et al,[33] Jaffe et al,[35] Conrad et al,[41] Takahata et al[34] |
| | Seroprotection anti-Hbs >10 mIU/ml | Conversion from non-protective to protective | Palazzo et al[44] |
| **Measles** | Absolute titer ≥8.0 IU/ml | Conversion from non-protective to protective | Aoki et al[36] |
| | Absolute titer ≥4.0 IU/ml | | Kawamura et al[37] |
| **Mumps** | Absolute titer ≥6.0 IU/ml | Conversion from non-protective to protective | Aoki et al[36] |
| | Absolute titer ≥4.0 IU/ml | | Kawamura et al[37] |
| **Rubella** | Absolute titer ≥8.0 IU/ml | Conversion from non-protective to protective | Aoki et al[36] |
| | Absolute titer ≥4.0 IU/ml | | Kawamura et al[37] |
| **Varicella zoster virus** | | Conversion from non-protective to protective Or ≥4-fold rise in titer | Camargo et al,[38] Stadtmauer et al[39] |

**Table S5.** Suggestions for evaluation of vaccine response in clinical practice. .

| Vaccine | Cut-off value for response |
|---|---|
| Pneumococcus | ≥0.35 ug/ml |
| Meningococcus | ≥1.0 ug/ml |
| Hepatitis B | >10 mIU/ml |
| Measles | ≥0.15 IU/ml |
| Tetanus | ≥0.1 IU/ml |
